# Effects of Unilateral Eccentric versus Concentric Training of Nonimmobilized Arm during Immobilization

**DOI:** 10.1249/MSS.0000000000003140

**Published:** 2023-02-18

**Authors:** TREVOR C. CHEN, SHANG-HEN WU, HSIN-LIAN CHEN, WEI-CHIN TSENG, KUO-WEI TSENG, HSING-YU KANG, KAZUNORI NOSAKA

**Affiliations:** 1 Department of Physical Education and Sport Sciences, National Taiwan Normal University, Taipei City, TAIWAN; 2 Department of Physical Education, Health and Recreation, National Chiayi University, Chiayi County, TAIWAN; 3 Department of Physical Education, University of Taipei, Taipei City, TAIWAN; 4 Department of Exercise and Health Sciences, University of Taipei, Taipei City, TAIWAN; 5 Centre for Human Performance, School of Medical and Health Sciences, Edith Cowan University, Joondalup, WA, AUSTRALIA

**Keywords:** CROSS-EDUCATIONAL EFFECT, MUSCLE CROSS-SECTIONAL AREA, MUSCLE HARDNESS, DELAYED-ONSET MUSCLE SORENESS, MAXIMAL VOLUNTARY CONTRACTION, CREATINE KINASE

## Abstract

**Introduction:**

The present study tested the hypothesis that eccentric training (ET) of nonimmobilized arm would attenuate negative effects of immobilization and provide greater protective effects against muscle damage induced by eccentric exercise after immobilization, when compared with concentric training (CT).

**Methods:**

Sedentary young men were placed to ET, CT, or control group (*n* = 12 per group), and their nondominant arms were immobilized for 3 wk. During the immobilization period, the ET and CT groups performed five sets of six dumbbell curl eccentric-only and concentric-only contractions, respectively, at 20%–80% of maximal voluntary isometric contraction (MVCiso) strength over six sessions. MVCiso torque, root-mean square (RMS) of electromyographic activity during MVCiso, and bicep brachii muscle cross-sectional area (CSA) were measured before and after immobilization for both arms. All participants performed 30 eccentric contractions of the elbow flexors (30EC) by the immobilized arm after the cast was removed. Several indirect muscle damage markers were measured before, immediately after, and for 5 d after 30EC.

**Results:**

ET increased MVCiso (17% ± 7%), RMS (24% ± 8%), and CSA (9% ± 2%) greater (*P* < 0.05) than CT (6% ± 4%, 9% ± 4%, 3% ± 2%) for the trained arm. The control group showed decreases in MVCiso (−17% ± 2%), RMS (−26% ± 6%), and CSA (−12% ± 3%) for the immobilized arm, but these changes were attenuated greater (*P* < 0.05) by ET (3% ± 3%, −0.1% ± 2%, 0.1% ± 0.3%) than CT (−4% ± 2%, −4% ± 2%, −1.3% ± 0.4%). Changes in all muscle damage markers after 30EC were smaller (*P* < 0.05) for the ET and CT than the control group, and ET than the CT group (e.g., peak plasma creatine kinase activity: ET, 860 ± 688 IU·L^−1^; CT, 2390 ± 1104 IU·L^−1^; control, 7819 ± 4011 IU·L^−1^).

**Conclusions:**

These results showed that ET of the nonimmobilized arm was effective for eliminating the negative effects of immobilization and attenuating eccentric exercise–induced muscle damage after immobilization.

Muscle disuse decreases muscle mass, muscle cross-sectional area (CSA), and force-generating capacity (1). Wall et al. (2) reported that immobilization by a full-leg cast for 5 d already reduced maximal voluntary isometric contraction (MVCiso) torque of the knee extensors (−9.0% ± 2.3%) and quadriceps CSA (−3.5% ± 0.5%). A longer period of immobilization (4–6 wk) could lead to greater decreases in muscle CSA for the elbow flexors (−11%) (3) and extensors (20%–32% (4), knee extensors (−16%), and soleus (−17%) as well as gastrocnemius (−26%) muscles (5).

It is well known that muscle strength gain conferred by a unilateral limb resistance training is transferred to a nontrained homologous muscle of the contralateral limb, which is referred to as the cross-education effect (6–8). A meta-analysis study by Munn et al. (8) showed that the magnitude of increase in muscle strength of the contralateral limb was 35% (95% confidence interval (CI), 20.9%–49.3%) of that of the ipsilaterally trained limb. Green and Gabrial (7) revealed that the cross-education effect was similar between upper- and lower-limb muscles, between sexes, and between young and old individuals, and the ratio between the nontrained and trained muscle strength gain ranged between 48% and 77% among 96 studies. Importantly, the magnitude of the cross-education effect seems to be greater after eccentric (ET) than concentric resistance training (CT). Tseng et al. (9) reported that a 5-wk progressive ET of the elbow flexors using a dumbbell of 10%, 30%, 50%, 80%, and 100% of MVCiso strength of the elbow flexors increased MVCiso strength of the trained arm (19% ± 8%) and untrained arm (11% ± 5%) greater than CT (10% ± 6% and 5% ± 2%, respectively) over the 5-wk period. Andrushko et al. (10) showed that muscle strength and CSA of immobilized wrist flexors for 4 wk were preserved by ET performed three times a week by the nonimmobilized arm (−2.4%, 1.3%) when compared with the control group without any training (−21.6%, −2.3%). Valdes et al. (11) showed that a 4-wk sling immobilization (8 h·d^−1^) of the elbow flexors decreased upper arm circumference (CIR) by 5% and MVCiso torque by 22%, but these decreases were attenuated by concentric–eccentric coupled resistance training (no decrease in MVCiso torque, 2.1% decrease in arm circumference) that were performed three times a week by the nonimmobilized arm. Interestingly, they reported that MVCiso torque of the elbow flexors increased 12% in the immobilized arm when the nonimmobilized arm performed eccentric-only resistance training with a heavier dumbbell (11).

It is also important to consider the responses of the immobilized muscles to resistance training after immobilization. It is well documented that eccentric exercise induces muscle damage that is characterized by delayed-onset muscle soreness, prolonged decreases in muscle function, and increased muscle hardness (or muscle stiffness) when it is performed for the first time or no exposure to eccentric contractions for a long period (12,13). Chen et al. (14) showed that the magnitude of muscle damage induced by maximal eccentric exercise of the elbow flexors was reduced when the second bout of the same exercise was performed by the contralateral homologous muscle. For instance, average changes in indirect muscle damage markers such as MVCiso torque, range of motion (ROM), CIR, muscle soreness, and plasma creatine kinase (CK) activity were smaller after the second bout of the eccentric exercise performed by the contralateral arm at 1 d (51%), 7 d (48%), and 28 d (26%) after the first bout. Thus, it seems possible that resistance training of the nonimmobilized arm could attenuate the magnitude of muscle damage of the immobilized arm when eccentric exercise is performed after the immobilization. However, to the best of our knowledge, no previous study has compared the effects of ET versus CT of the nonimmobilized arm on muscle damage of the immobilized arm.

Therefore, the present study compared the effects of ET versus CT of the elbow flexors of the nonimmobilized arm on muscle function and CSA of the immobilized arm by a cast for 3 wk, and on muscle damage of the immobilized arm that performed eccentric exercise of the elbow flexors after immobilization. We hypothesized that ET would attenuate decreases in muscle function and CSA of the immobilized arm greater than CT, and ET would provide greater protective effects against muscle damage induced by maximal eccentric exercise after immobilization than CT.

## METHODS

### Participants and Study Design

Thirty-six sedentary healthy young men who had no musculoskeletal injuries of arms were recruited for this study. Each of them provided an informed consent to participate in the study that had been approved by the Research Ethic Committee of National Taiwan Normal University. The study was conducted in conformity with the policy statement regarding the use of human participants by the Medicine & Science in Sports & Exercise® and Declaration of Helsinki.

The sample size was estimated using the data from our previous study in which the contralateral repeated bout effect of the nontrained elbow flexors after a 5-wk progressive ET and CT was compared (9). Based on the effect size of 1.2 for the difference in the increase in MVCiso torque of the elbow flexors between the ET (11% ± 6%) and CT (5% ± 4%) groups, with an *α* level of 0.05, and a power (1 − *β*) of 0.80 in the one-tailed dependent *t*-test, it was estimated that at least 10 participants were necessary per group (G*Power 3.1.9.2, Heinrich-Heine-Universitat Dusseldorf, Dusseldorf, Germany; http://www.gpower.hhu.de/). Thus, considering possible dropouts and estimation error, 12 participants for each group were recruited in the present study. Because the previous study (9) used male participants only, and possible sex differences in responses to muscle damage induced by eccentric exercise were documented (15,16), the present study used only male participants. Their mean (±SD) age, height, body mass, and body mass index of the 36 participants were 22.7 ± 1.7 yr, 172.4 ± 6.5 cm, 73.7 ± 11.2 kg, and 24.8 ± 3.8 kg·m^−2^, respectively.

A familiarization session was set at 3–5 d before the immobilization. The participants experienced all measurements shown hereinafter. MVCiso torque was measured from both arms after warm-up exercise and some practice trials. Based on the MVCiso torque, the participants were placed into one of the three groups (*n* = 12 per group); control, ET, and CT groups by matching the average baseline MVCiso torque among the groups as much as possible. No significant differences in the age, height, body mass, and body mass index were evident among the groups.

**FIGURE 1:**
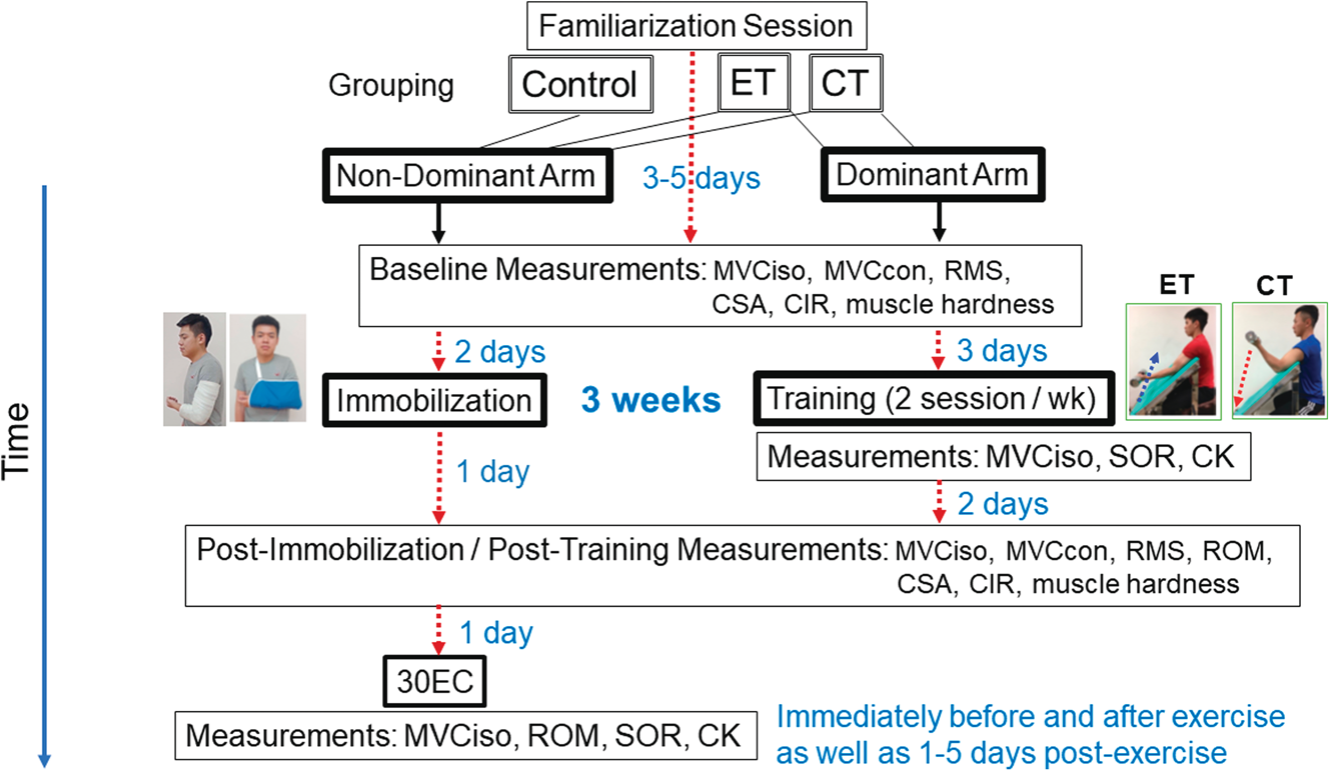
Experimental design and testing procedures of the study. The nondominant arms of the participants in the control, ET, and CT groups were immobilized for 3 wk, whereas the dominant arms of the participants of the ET and CT groups performed eccentric-only and concentric-only training of the elbow flexors twice a week during the 3-wk immobilization period. The nondominant arms performed 30 maximal eccentric contractions of the elbow flexors (30EC) after the immobilization. Multiple measurements were taken before and after immobilization from both arms, before and after each training session from the dominant arm, and before and after 30EC from the nondominant arm. CC, concentric contractions; CSA, biceps brachii cross-sectional area; MVCcon, maximal concentric contraction torque of the elbow flexors and extensors at 30°·s^−1^; MVCiso, maximal isometric contraction strength and torque at 90° of elbow flexion; RMS, root-mean square of surface electromyographic activity during MVCiso; SOR, muscle soreness.

As shown in Figure 1, all participants received immobilization of the nondominant arm (opposite to the arm that could throw a ball better, faster, and further) for 3 wk as explained hereinafter. The participants in the ET and CT groups performed elbow flexor exercise with the nonimmobilized (dominant) arm twice a week during the 3-wk immobilization period as detailed hereinafter. All participants performed maximal elbow flexor eccentric exercise of the immobilized arm at 2 d after the immobilization.

### Immobilization

The nondominant arm was immobilization by a cast at the elbow joint of 90° flexion and secured in a sling with a mild shoulder internal rotation to unload the elbow flexor muscles (Fig. 1). This protocol was adapted and modified from previous studies that showed 13%–16% decreases in MVCiso strength of the elbow flexors after a 2-wk immobilization of upper arm (17,18). The participants were instructed not to remove the sling except when changing their clothes, bathing, and sleeping.

### Training during Immobilization

Each training session consisted of five sets of six eccentric-only or concentric-only contractions of the elbow flexors using a dumbbell. In the ET, each participant was instructed to lower a dumbbell from an elbow flexed (90°) to a fully extended position (0°) in 3 s, and the investigator removed the dumbbell at the extended position, and the arm was returned to the start position without a dumbbell. In the CT, each participant was instructed to lift a dumbbell from an elbow extended (≈5°) to a flexed position (90°) in 3 s. After each concentric contraction, the arm was returned to the start position without a dumbbell, and the investigator spotted a participant if he showed difficulty at long muscle lengths during the last training session in which a heavy weight was used. The interval was 15 s between contractions and 2 min between sets based on our previous study (9).

To determine the dumbbell weight for the progressive ET or CT, MVCiso strength of the unilateral elbow flexors at 90° elbow flexion was measured by a loadcell (model DFG51; Omega Engineering, Stamford, CT) that was attached to a cuff surrounding the wrist of the exercise arm, according to our previous study (9). It should be noted that MVCiso strength is normally smaller than eccentric one-repetition maximum (1RM) strength but greater than concentric 1RM strength, and the MVCiso strength at 90° elbow flexion is close to maximal eccentric and concentric strength at extended (<30° flexion) elbow joint angles (19). Because as few as two maximal eccentric contractions could attenuate the magnitude of muscle damage induced by the subsequent bout of maximal eccentric contractions of the same or opposite limb homologous muscle (20), the load for ET and CT was determined by the MVCiso strength not by 1RM test. Each participant was seated on a custom-made preacher curl bench, placing the elbow joint angle at 90° and the shoulder joint angle at 45° flexion and 0° abduction. The participant was instructed to flex the elbow joint maximally for 3 s, and this was repeated three times with a 45-s rest between attempts. The highest value of the three peak values was used to determine the dumbbell weight (21).

**FIGURE 2:**
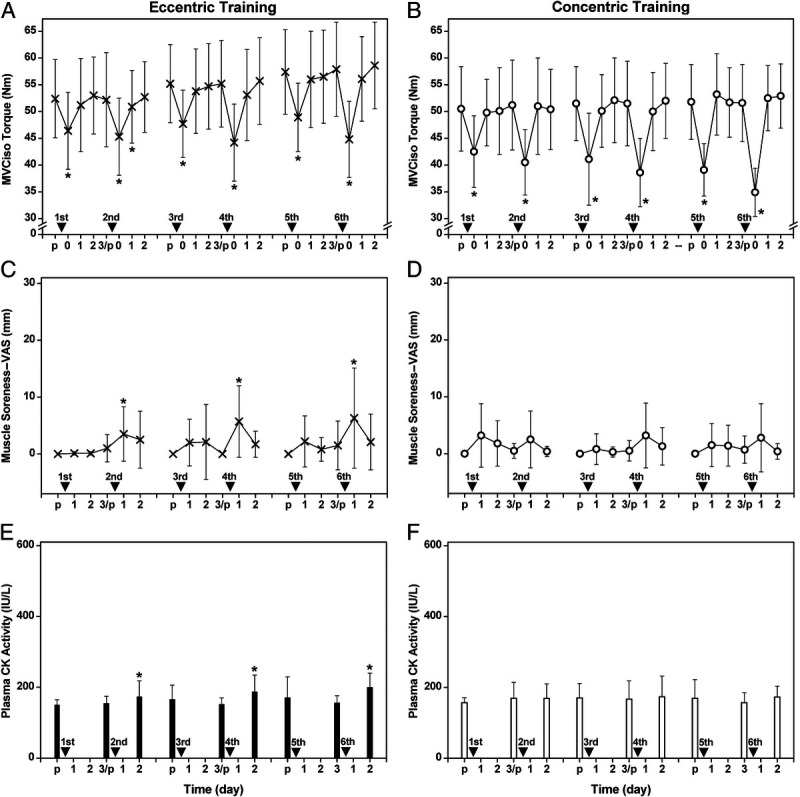
Changes (mean ± SD) in MVCiso torque (A, B), muscle soreness assessed by a 100-mm visual analog scale (C, D), and plasma CK activity (E, F) activity before (p), immediately after (0), and 1–2 or 1 to 3 d after the first (20%), second (40%), third (40%), fourth (60%), fifth (60%), and sixth (80%) training sessions for the ET group (*left*) and CT group (*right*). *Significant difference (*P* < 0.05) from the pretraining value.

The ET and CT protocols were modified from our previous study (9) showing that minimal muscle damage was induced in a 5-wk progressive eccentric resistance exercise training. The load for each exercise session in the present study was increased by 20%, 40%, 40%, 60%, 60%, and 80% of the MVCiso strength that was reassessed at each week for both ET and CT (9). The training was performed twice a week with a 3-d rest between sessions. Changes in MVCiso torque and muscle soreness of the trained arm, and plasma CK activity were measured before, and 1–2 or 1–3 d after each training session (Fig. 2).

### Eccentric Exercise

All participants performed five sets of six eccentric contractions (30EC) of the elbow flexors of the immobilized arm with a dumbbell corresponding to the MVCiso strength. Each participant was instructed to lower a dumbbell from an elbow flexed (90°) to an elbow fully extended position (0°) in 3 s, and the investigator removed the dumbbell at the extended position, and the arm was returned to the start position without load. The eccentric contraction was repeated every 15 s, and a 2-min rest was inserted between sets (9).

### Dependent Variables

The dependent variables consisted of MVCiso torque of the elbow flexors and MVCcon torque of the elbow flexors and extensors, root-mean square (RMS) during MVCiso, ROM, biceps brachii CSA by ultrasound extended-field-of-view (EFOV) imaging, CIR, muscle hardness, muscle soreness, and plasma CK activity. Among them, MVCiso torque, muscle soreness, and plasma CK activity were taken before, immediately after, and 1–2 or 1–3 d after each training session to monitor muscle damage for the ET and CT groups (Fig. 1). To assess the effects of immobilization on the immobilized and nonimmobilized arms, MVCiso and MVCcon torques, RMS, CSA, CIR, and muscle hardness were measured before and at 1 d after immobilization. To assess muscle damage after eccentric exercise for the immobilized arm, MVCiso torque, ROM, muscle soreness, and plasma CK activity were assessed before, immediately after (only for MVCiso torque and ROM), and 1, 2, 3, 4, and 5 d after the exercise from the exercised arm.

#### MVCiso and MVCcon torques

The MVCiso and MVCcon torque measurements were the same as those of our previous study; thus, the details can be found in elsewhere (9,22). Briefly, the MVCiso torque was measured at 90° (1.57 rad) elbow flexion, where the full elbow extension angle was considered as 0° (0 rad) using a dynamometer (Biodex System S4; Biodex Medical Systems, Shirley, NY). The MVCcon torque was measured at the angular velocity of 30°·s^−1^ (0.52 rad·s^−1^) for the ROM of 120° (2.09 rad) for the elbow flexors (0°–120°) and extensors (120°–0°) for three continuous contractions for both directions by the isokinetic dynamometer, in the same position as that of the MVCiso torque measure. The MVCiso torque was measured first followed by the MVCcon torque measures with a 5-min rest between the MVCiso and MVCcon measures for each arm. Verbal encouragement was provided during the tests. The highest value of the three trials was used for further analysis of the MVCiso torque as well as the MVCcon torque of the elbow flexors (EF-MVCcon) and extensors (EE-MVCcon) (14).

#### Surface electromyography

Muscle activity was recorded using the Ultium EMG sensor system (Noraxon, INC, Scottsdale, AZ) with disposable Ag/AgCl pregelled electrodes during MVCiso measurements. The participants’ skin was cleaned thoroughly and prepared before electrode placement. The surface electrodes were attached to the belly of the biceps brachii (2-cm center-to-center interelectrode distance). Raw EMG signals during the MVCiso measures were sampled with a frequency of 2000 Hz using a band-pass filter (10–500 Hz), and RMS was obtained for a 500-ms slot in the plateau MVCiso torque during the 3-s MVCiso (23). The average of peak RMS from three MVCiso trials on each time point was used for further analyses.

#### Range of motion

The ROM of the elbow joint was determined as the difference between the elbow joint angles of maximal voluntarily flexion and extension measured by a manual goniometer (9,24). Three measurements were taken for each angle, and the mean of the three measurements was used to calculate ROM (9,24).

#### Bicep brachii CSA

Using the EFOV method that was adapted from the previous study (25), biceps brachii was captured at 50% distal between the lateral epicondyle of the humerus and the acromial process of the shoulder. Each participant was lay supine in a comfortable position with arms fully resting on a massage bed. Pressure was applied minimally but consistently avoiding compression of the muscle, and transmission gel was applied to aid in acoustic coupling. EFOV scans were obtained using the same real-time B-mode ultrasound apparatus (Aloka Prosound α6 ultrasound system; Hitachi Co. Ltd., Tokyo, Japan) with a 7.5-MHz 4.0-cm probe (UST-5412) by moving the probe along the marked lines axially from the medial aspect to the lateral aspect of the upper arm in a continuous single view by the same investigator. Scanning velocity was controlled to allow clear EFOV images, and care was taken to avoid exerting too much pressure on the skin surface. Two scans were taken from each region, and the biceps brachii muscle was traced to calculate its CSA using a computer software program (ImageJ, version 0.0; National Institutes of Health, Bethesda, MD). Because some of the images did not include the whole area of brachialis clearly, only the biceps brachii CSA was obtained in the present study.

#### Upper arm circumference

While each participant was standing, relaxing, and letting the arm hang down by his side, the upper arm CIR was measured at the midportion of the upper arm, between the acromion process of the clavicle and the lateral epicondyle of the humerus, using a Gulick tape measure (Creative Health Products, Plymouth, MI). The measurements were taken three times by the same examiner, and the mean of the three measures was used for statistical analysis (9).

#### Muscle hardness

Muscle hardness was measured by a tissue hardness algometer (OE-220; Ito Co. Ltd., Tokyo, Japan) at the midportion of the biceps brachii (as the same site as that for the CSA measure) when each participant lay supine with the testing arm was fully extended and relaxed on a message bed. Muscle hardness was measured three times at each time point, and the mean value of the three was calculated and used for further analysis (26).

#### Muscle soreness

Muscle soreness was quantified using a visual analog scale that had a 100-mm continuous line with “not sore at all” on one side (0 mm) and “very, very sore” on the other side (100 mm). The investigator asked the participant to rate his perceived soreness on the visual analog scale when the muscles were passively extended for the ROM (120°–0° of elbow flexion angles) measures (9,27).

#### Plasma CK activity

Approximately 5 mL of venous blood was withdrawn by a standard venipuncture technique from the cubital fossa region of the arm and centrifuged for 10-min to extract plasma, and plasma samples were stored at −80°C until analyses. Plasma CK activity was assayed spectrophotometrically by an automated clinical chemistry analyzer (Model 7080; Hitachi, Co. Ltd., Tokyo, Japan) using a commercially available test kit (Roche Diagnostics, Indianapolis, IN) (9,28).

#### Test–retest reliability of the measures

The test–retest reliability of the dependent variables except muscle hardness indicated by the coefficient of variation was shown to be smaller than 9.9% in the previous studies performed in the same laboratory and by the same investigators (9,25). Coefficient of variation of the muscle hardness measure that was assessed in our laboratory was 9.6%.

### Cross-Education Effect

The cross-education effect ratio was calculated as the ratio between the trained and nontrained arms for the changes in MVCiso and MVCcon torques, RMS, CSA, CIR, and muscle hardness from pretraining to posttraining for each participant in the ET and CT groups by the following formula based on the previous study (29).

Cross-education effect ratio (%) = (change in the nontrained arm from pretraining to posttraining/change in the trained arm from pretraining to posttraining) × 100.

In addition, the difference in each variable changes over the 3-wk immobilization period between the nonimmobilized and immobilized arms was examined to compare the magnitude of the cross-education effect among the three groups. The relationships between the changes in the variables in the nonimmobilized arm and those in the immobilized arm were also examined for the participants in each group.

### Statistical Analyses

Shapiro–Wilk test was used to verify the normality assumption of the data, which showed that normality assumption was met for all variables in the present study. Baseline values of all dependent variables before the 3-wk immobilization were compared among the ET, CT, and control groups by a one-way ANOVA for the immobilized arm and nonimmobilized arm, separately. A mixed-design two-way ANOVA (group [3] × time [2]) was used to compare the three groups for changes in each dependent variable before and after immobilization. When this showed a significant (*P* < 0.05) interaction effect, a mixed-design two-way ANOVA (group [2] × time [2]) was followed to compare between two groups (i.e., ET and CT, ET and control, and CT and control) for the changes in the dependent variables. When the ANOVA showed a significant interaction effect, a Tukey’s *post-hoc* test was performed. To compare changes in muscle damage markers before, immediately after and 1–5 d after 30EC among the groups, a mixed-design two-way ANOVA (group [3] × time [6 or 7]) was performed. When a significant interaction effect was evident, a mixed-design two-way ANOVA was also performed to compare the changes between the ET and CT, ET and control, and CT and control groups, respectively (group [2] × time [6 or 7]). When the ANOVA found a significant interaction effect, a Tukey’s *post-hoc* test was performed. A one-way ANOVA was used to compare the magnitude of the training effect, cross-education effect, and contralateral repeated bout effect on each variable between the ET and CT groups. When the ANOVA found a significant main effect, a *t*-test was followed as a *post-hoc* test. *η*^2^ Values were calculated as measures of effect size when necessary, and they were considered that a value of ~0.02 is a small effect; ~0.13, a medium effect; and >0.26, a large effect (30,31). Pearson product-moment correlation coefficient (*r*) was used to examine the relationships between the nonimmobilized and immobilized arms for the normalized changes in MVCiso and MVCcon of the elbow flexors, RMS during EF-MVCiso, CSA, CIR, and muscle hardness for the ET, CT, and control groups (*n* = 12 per group), separately. The difference in each variable changes over the 3-wk immobilization period between the nonimmobilized and immobilized arms was compared among the ET, CT, and control groups by one-way ANOVA. A significant level was set at *P* ≤ 0.05. The data were presented as mean ± SD, unless otherwise stated.

## RESULTS

### Baseline measurements

No significant differences (*P* > 0.05) in the baseline values of any of the dependent variables were found before the immobilization or training among the groups for the immobilized and nonimmobilized arms (Table 1).

**TABLE 1:** Changes (mean ± SD) in MVCiso torque of the elbow flexors and maximal voluntary concentric contraction torque of the elbow flexors (EF-MVCcon) and extensors (EE-MVCcon), RMS of surface electromyographic activity of biceps brachii during MVCiso, biceps brachii CSA, CIR, and muscle hardness (hardness) before (Pre) and after a 3-wk immobilization (Post) for the nonimmobilized arm and immobilized arm for the control group, and ET and CT groups that performed training with the nonimmobilized arm during the immobilization.

	Nonimmobilized Arm							Immobilized Arm						
	Control		ET		CT		*F*_2,3_ *P*	Control		ET		CT		*F*_2,3_ *P*
	Pre	Post	Pre	Post	Pre	Post		Pre	Post	Pre	Post	Pre	Post	
MVCiso (N·m)	51.3 ± 8.2	51.7 ± 8.7	52.4 ± 7.3	61.0 ± 8.6*^,^**^,^***	50.5 ± 7.9	53.6 ± 8.3*	164.4 <0.001	49.7 ± 8.5	41.4 ± 6.7*	51.3 ± 5.7	53.1 ± 5.5*^,^**^,^***	49.2 ± 7.9	47.2 ± 8.1*^,^**	36.0 <0.001
EF-MVCcon (N·m)	33.1 ± 3.9	33.2 ± 4.3	35.9 ± 2.5	40.8 ± 2.2*^,^**^,^***	33.9 ± 3.5	36.3 ± 4.1*	44.9 <0.001	32.4 ± 6.1	26.1 ± 4.3*	34.4 ± 3.0	36.0 ± 3.1*^,^**^,^***	31.9 ± 3.7	30.7 ± 3.3*^,^***	121.6 <0.001
EE-MVCcon (N·m)	31.9 ± 3.7	32.0 ± 4.0	33.0 ± 2.7	34.5 ± 2.6	31.1 ± 3.2	31.5 ± 3.6	1.9 0.163	30.9 ± 6.8	27.2 ± 5.8*	31.9 ± 3.0	32.2 ± 2.9*^,^**^,^***	29.7 ± 3.2	29.0 ± 3.0*	85.0 <0.001
RMS (μV)	1457 ± 576	1438 ± 546	1485 ± 308	1828 ± 364*^,^**^,^***	1376 ± 240	1491 ± 251*	66.1 <0.001	1392 ± 540	1018 ± 361*	1353 ± 493	1620 ± 354*^,^**^,^***	1303 ± 185	1241 ± 182*^,^**	52.6 <0.001
CSA (cm^2^)	7.33 ± 0.97	7.35 ± 0.93	7.39 ± 1.92	8.04 ± 1.98*	7.54 ± 1.75	7.75 ± 1.76*	32.1 <0.001	7.31 ± 0.87	6.41 ± 0.66*	7.26 ± 1.81	7.25 ± 1.85**	7.35 ± 1.12	7.06 ± 1.12*	80.7 <0.001
CIR (mm)	302.0 ± 33.8	301.8 ± 31.6	301.2 ± 38.8	311.7 ± 39.5*	300.2 ± 51.9	304.1 ± 52.1*	15.5 <0.001	298.5 ± 29.2	287.8 ± 29.5*	296.4 ± 35.2	296.8 ± 35.2	297.5 ± 41.0	293.6 ± 41.0*	115.3 <0.001
Hardness (N·cm^−2^)	33.2 ± 2.8	33.4 ± 3.0	32.7 ± 3.7	30.4 ± 3.1*^,^**	32.4 ± 3.4	33.2 ± 4.0	22.0 <0.001	33.6 ± 3.4	40.1 ± 3.5*	32.1 ± 5.3	31.1 ± 5.6*^,^**	31.3 ± 5.0	33.4 ± 5.2*^,^**	118.4 <0.001

*F* and *P* values of the interaction effect among the three groups based on the mixed-design two-way ANOVA are shown on the right column of each arm.

*Significant (*P* < 0.05) difference from the Pre value.

**Significant (*P* < 0.05) difference from the control group.

***Significant (*P* < 0.05) difference from the CT group based on *post-hoc* test (*t*-test).

### Training

All participants in the ET and CT groups performed the six training sessions as planned without missing any session. As shown in Figure 2, small but significant (*P* < 0.05) changes in MVCiso torque, muscle soreness, and plasma CK activity were observed after ET. In contrast, significant (*P* < 0.05) changes were evident only for MVCiso torque immediately after CT.

### Effects of training on the trained (nonimmobilized) arm

The control group showed no significant (*P* > 0.05) changes in the variables for the nonimmobilized arm (Table 1). A significant (*P* < 0.05) interaction effect among the control, ET, and CT groups was evident for each variable. The ET and CT groups showed significant (*P* < 0.05) increases in MVCiso torque and EF-MVCcon, RMS of the biceps brachii during MVCiso, biceps brachii CSA, and CIR for the trained arm, but a decrease in muscle hardness (*P* = 0.001) was found for the ET only. When comparing between the ET and CT groups, changes in all measures except for EE-MVCcon in the nonimmobilized (trained) arm were greater (*P* ≤ 0.001) for the ET than CT group (interaction effect: MVCiso: *F*_1,22_ = 21.3, *η*^2^ = 0.492; EF-MVCcon: *F*_1,22_ = 182.3, *η*^2^ = 0.892; RMS: *F*_1,22_ = 37.8, *η*^2^ = 0.632; CSA: *F*_1,22_ = 75.9, *η*^2^ = 0.775; CIR: *F*_1,22_ = 15.5, *η*^2^ = 0.398; hardness: *F*_1,22_ = 28.8, *η*^2^ = 0.567).

### Effects of training on the immobilized (nontrained) arm

A significant interaction effect among the three groups was evident for each variable (Table 1). The control group showed significant (*P* < 0.05) decreases in MVCiso, EF-MVCcon and EE-MVCcon torques, RMS of the biceps brachii during MVCiso, biceps brachii CSA, and CIR, and an increase in muscle hardness. Significant changes in these measures (*P* < 0.05) were also evident for the CT group, but the magnitude of the changes in MVCiso and EF-MVCcon torques, RMS, and muscle hardness was smaller (*P* < 0.05) when compared with the control group. In contrast, the ET group showed significant (*P* < 0.05) increases in MVCiso and EF-MVCcon torques, and RMS during MVCiso; a decrease in muscle hardness; and no significant changes in CSA and CIR. When comparing with the control group, changes in all variables were significantly smaller (*P* < 0.001) for the ET group (interaction effect; MVCiso: *F*_1,22_ = 253.4, *η*^2^ = 0.920; EF-MVCcon: *F*_1,22_ = 111.0, *η*^2^ = 0.835; EE-MVCcon: *F*_1,22_ = 30.1, *η*^2^ = 0.578; RMS: *F*_1,22_ = 71.1, *η*^2^ = 0.764; CSA: *F*_1,22_ = 113.1, *η*^2^ = 0.837; CIR: *F*_1,22_ = 183.3, *η*^2^ = 0.893; hardness: *F*_1,22_ = 169.0, *η*^2^ = 0.885) and CT group (interaction effect; MVCiso: *F*_1,22_ = 96.3, *η*^2^ = 0.814; EF-MVCcon: *F*_1,22_ = 22.4, *η*^2^ = 0.504; EE-MVCcon: *F*_1,22_ = 67.7, *η*^2^ = 0.755; RMS: *F*_1,22_ = 27.8, *η*^2^ = 0.558; CSA: *F*_1,22_ = 58.8, *η*^2^ = 0.728; CIR: *F*_1,22_ = 60.0, *η*^2^ = 0.732; hardness: *F*_1,22_ = 61.5, *η*^2^ = 0.737). When comparing the ET and CT groups, the changes were significantly smaller (*P* < 0.001) for the ET than CT group (interaction effect; MVCiso: *F*_1,22_ = 106.6, *η*^2^ = 0.829; EF-MVCcon: *F*_1,22_ = 19.0, *η*^2^ = 0.464; EE-MVCcon: *F*_1,22_ = 116.3, *η*^2^ = 0.841; RMS: *F*_1,22_ = 74.1, *η*^2^ = 0.771; CSA: *F*_1,22_ = 37.1, *η*^2^ = 0.628; CIR: *F*_1,22_ = 98.7, *η*^2^ = 0.818; hardness: *F*_1,22_ = 144.0, *η*^2^ = 0.867).

### Cross-education effects

**FIGURE 3:**
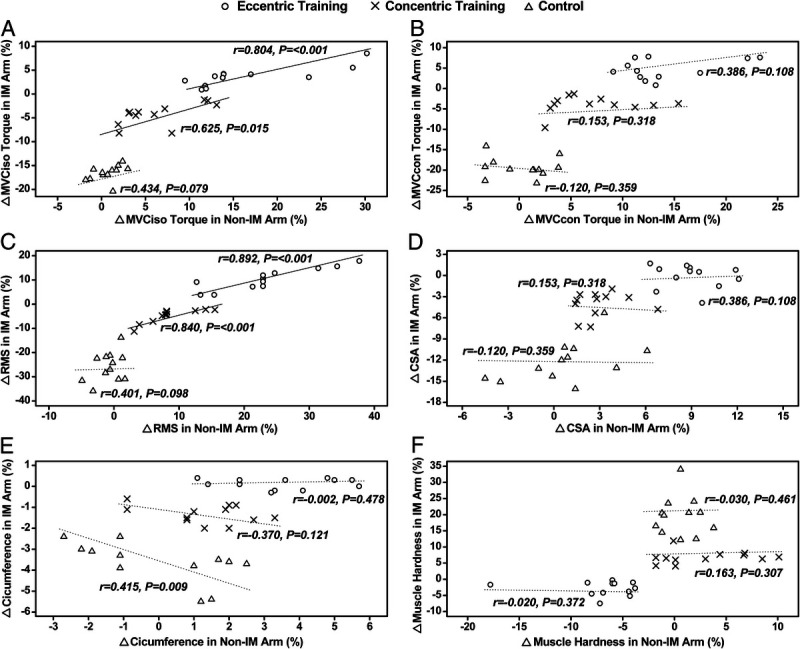
Correlations between the nonimmobilized (Non-IM) and immobilized (IM) arms for the magnitude of changes in maximal voluntary isometric (MVCiso; A) and concentric contraction torque of the elbow flexors (MVCcon; B), RMS of surface electromyographic activity during MVCiso (RMS; C), biceps brachii cross-sectional area (CSA; D), CIR (E), and muscle hardness (F) over 3-wk immobilization period for the ET (shown by ×), CT (shown by ○), and control (shown by △) group (*n* = 12 per group), respectively. A solid line represents a significant (*P* < 0.05) correlation, and a dotted line represents a nonsignificant (*P* > 0.05) correlation.

The cross-education effect ratio was significant (*P* ≤ 0.001) for MVCiso torque (21.7% ± 8.3%), EF-MVCcon torque (36.8% ± 20.4%), RMS (43.5% ± 12.7%), and CIR (6.1% ± 11.8%) in the ET group only. As shown in Figure 3, a significant (*P* < 0.02) correlation between the nonimmobilized (trained) and immobilized arms was found in the ET and CT groups for the changes in MVCiso (ET: *r* = 0.804, CT: *r* = 0.625) and RMS (ET: *r* = 0.892, CT: *r* = 0.840) only.

### Changes in muscle damage markers after 30EC

**FIGURE 4:**
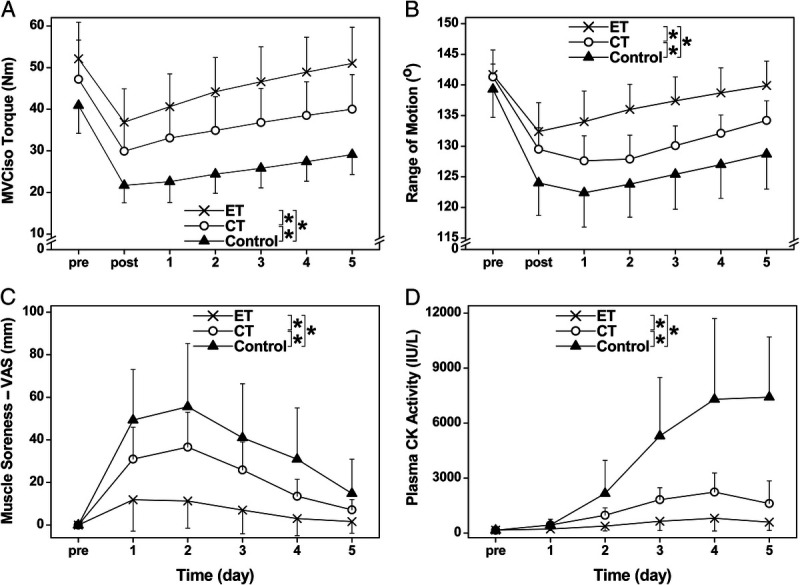
Changes (mean ± SD) in MVCiso torque (A), ROM (B), and changes in muscle soreness by 100-mm visual analog scale (C), and plasma CK activity (D) from the baseline (pre) at immediately (0), and 1, 2, 3, 4, and 5 d after maximal eccentric exercise of the elbow flexors for the control, ET, and CT groups. *Significant (*P* < 0.05) interaction effect by mixed-design two-way ANOVA for the corresponding two groups.

All groups showed significant (*P* < 0.05) changes in MVCiso torque, ROM, muscle soreness, and plasma CK activity after 30EC performed by the immobilized arm (Fig. 4). A significant interaction effect (*P* < 0.05) was evident for changes in MVCiso torque (*F*_12,198_ = 14.2, *η*^2^ = 0.462), ROM (*F*_12,198_ = 28.7, *η*^2^ = 0.635), muscle soreness (*F*_10,165_ = 10.4, *η*^2^ = 0.387), and plasma CK activity (*F*_10,165_ = 28.5, *η*^2^ = 0.633) over time among the three groups. When compared with the control group, the changes were significantly smaller for the ET group (interaction effect; MVCiso: *F*_6,132_ = 27.0, *P* < 0.001, *η*^2^ = 0.551; ROM: *F*_6,132_ = 66.4, *P* < 0.001, *η*^2^ = 0.751; muscle soreness: *F*_5,110_ = 18.1, *P* < 0.001, *η*^2^ = 0.451; plasma CK activity: *F*_5,110_ = 35.5, *P* < 0.001, *η*^2^ = 0.618) and the CT group (MVCiso: *F*_6,132_ = 8.0, *P* < 0.001, *η*^2^ = 0.266; ROM: *F*_6,132_ = 5.4, *P* < 0.001, *η*^2^ = 0.198; muscle soreness: *F*_5,110_ = 3.8, *P* = 0.003, *η*^2^ = 0.147; CK: *F*_5,110_ = 23.6, *P* < 0.001, *η*^2^ = 0.517). The changes in the ET group were significantly (*P* < 0.001) smaller than those of the CT group for all variables (MVCiso: *F*_6,132_ = 7.1, *η*^2^ = 0.244; ROM: *F*_6,132_ = 26.4, *η*^2^ = 0.546; muscle soreness: *F*_5,110_ = 9.2, *η*^2^ = 0.296; CK: *F*_5,110_ = 11.2, *η*^2^ = 0.338).

**FIGURE 5:**
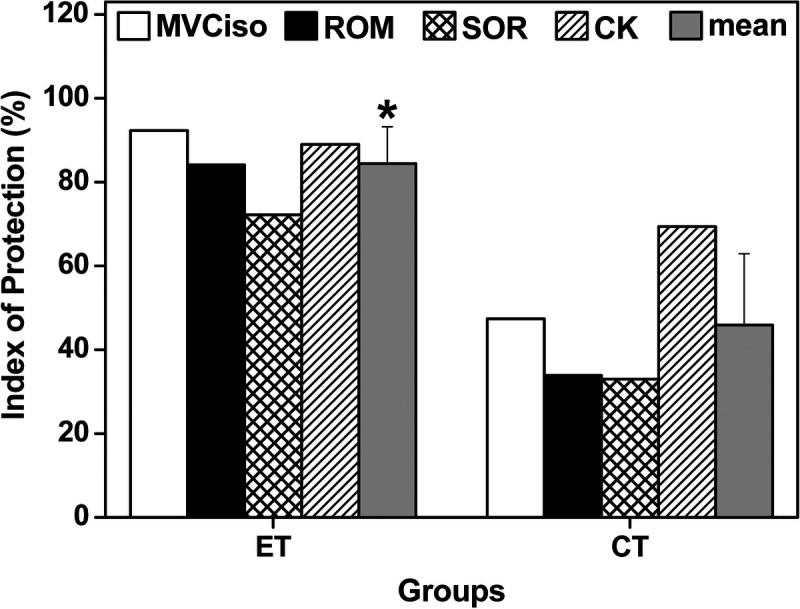
Index of protection for MVCiso torque of the elbow flexors, ROM, muscle soreness (SOR), and plasma CK activity, and the average and SD of the six variables (mean) for the ET and CT groups. The index was based on the comparison to the changes after the bout of the control group, which was calculated by the formula: [Change in the control condition − Change in ET or CT group]/[Change in the control group] × 100%. The “Change” in the formula refers to the magnitude of the change from the baseline at 5 d after exercise for MVCiso and ROM, and maximal change from the baseline for SOR and CK. *Significant (*P* < 0.05) difference from the CT group by a *t*-test.

Figure 5 compares the magnitude of the protective effect for each variable and the average of the four variables (MVCiso, ROM, muscle soreness, and plasma CK activity) between the ET and CT groups. The average protective effect from the values of the four variables was greater (*F*_1,5_ = 109.7, *P* < 0.001, *η*^2^ = 0.956) for the ET group (83.3% ± 13.9%) than the CT group (43.3% ± 17.4%).

## DISCUSSION

The results seem to support the hypotheses that 1) ET would attenuate decreases in neuromuscular function and CSA of the immobilized arm greater than CT, and 2) ET would provide greater protective effects against muscle damage induced by maximal eccentric exercise after immobilization than CT. In the sections hereinafter, hypotheses 1 and 2 are discussed, and practical applications and future research directions are provided.

### Effects of ET versus CT on trained and immobilized arms

The CT decreased MVCiso torque only at immediately after exercise, and no muscle soreness was developed after CT (Fig. 2). This suggests that no muscle damage was induced by CT, which was in line with the finding of previous studies (9,31). In ET, decreases in MVCiso torque lasted for 2 d after exercise, and increases in muscle soreness and plasma CK activity were observed after the second, fourth, and sixth training sessions. These indicate that muscle damage was induced at the higher-intensity (>40%) ET, but the magnitude of muscle damage was minor. Tseng et al. (9) also reported that the magnitude of muscle damage induced by eccentric exercise of the elbow flexors was minor, when the intensity of eccentric contractions was progressively increased from 10% to 100% of preexercise MVCiso level in a 5-wk ET.

No significant changes in any of the variables for the nonimmobilized arm were found for the control group, but the ET and CT groups showed significant changes in all variables after the 3-wk training for the nonimmobilized trained arm (Table 1). The normalized changes in all variables from baseline were greater for the ET than CT group. These results were consistent with the findings of previous training studies in which eccentric-only and concentric-only training were compared (9,29,32,33). These suggest that eccentric-only resistance training produces greater neuromuscular adaptations than concentric-only resistance training.

Regarding the changes in the variables of the immobilized arm, the control group showed decreases in MVCiso (−17%), EF-MVCcon (−19%), EE-MVCcon torque (−12%), RMS (−26%), CSA (−12%), and CIR (−4%) and an increase in muscle hardness (20%). Some of these changes were similar to those reported after a 3-wk sling immobilization (15 h·d^−1^) of the elbow flexors (1RM strength: −20%, muscle thickness: −6%) in a previous study (34). Thus, it seems that the magnitude of decreases in muscle strength and size in the control group was comparable to that normally induced by immobilization of the elbow flexors for the duration. These changes were smaller for the CT group, and importantly, the ET group showed increases in MVCiso (4%) and EF-MVCcon torque (5%) without changes in CSA (−0.1%) and CIR (0.1%), and a decrease in muscle hardness (−3%) (Table 1). Valdes et al. (11) reported that a 4-wk sling immobilization (8 h·d−^1^) of the elbow flexors decreased CIR by 5%, MVCiso torque by 22% and RMS during MVCiso by 35%, but these decreases were significantly attenuated by concentric–eccentric coupled resistance training (no decrease in MVCiso torque, 5.9% decrease in RMS, 2.1% decrease in arm circumference) that were performed three times a week by the nonimmobilized arm. Interestingly, they reported that MVCiso torque and RMS of the elbow flexors increased 12% and 17.5%, respectively in the immobilized arm when the nonimmobilized arm performed eccentric-only resistance training with a heavier dumbbell (11). The present study also found an increase in MVCiso torque and RMS after eccentric-only training for the nontrained (immobilization) arm, although the magnitude of the increase was smaller than that reported in the study by Valdes et al. (11). They speculated that eccentric-only training modulated the corticospinal and intracortical inhibition to a greater extent than the coupled concentric-ET. It may be that the 1RM tests performed by the trained arm during the ET and CT at before the first, third and fifth training sessions (9 tests in total) contributed to the cross-over effect (35,36). However, it is important to note that the same number of 1RM tests was performed by the ET and CT groups, thus the difference in the cross-education effect between the two groups cannot be explained by the 1RM tests.

It should be noted that no significant correlation between the nonimmobilized and immobilized arms was evident for the changes in the variables for the control group (Fig. 3). This may suggest that the immobilized arm was not influenced by the nonimmobilized arm without exercise training. A significant correlation between the trained and immobilized arms of the participants in the ET and CT groups was evident for the changes in MVCiso (ET: *r* = 0.804, CT: *r* = 0.625) and RMS (ET: *r* = 0.892, CT: *r* = 0.840) (Fig. 3). It appears that the greater the training effects, the greater the cross-education effect at least for MVCiso torque and its related variable (i.e., RMS during MVCiso). It is interesting that such a relationship was not observed for MVCcon, biceps brachii CSA, CIR and muscle hardness. This may be due to the small sample size, but it is puzzling how exactly the trained arm influenced the immobilized arm for some of the variables only. It may be that the mechanisms underpinning the cross-education effect on these variables are different from those on MVCiso. A significant positive cross-education effect ratio was evident only for the ET group (MVCiso: 22%, EF-MVCcon: 37%, RMS: 44%, CIR: 6%), suggesting that ET induces greater cross-education effect than CT. It has been reported that the magnitude of increase in muscle strength of the contralateral limb was 35% (95% CI: 20.9%–49.3%) (8) or 48%–77% of that of the ipsilaterally trained limb (7). Thus, it is likely that the greater cross-education effect by ET than CT was due to the greater training effects induced by ET than CT. It seems reasonable to conclude that to preserve muscle function and minimize muscle atrophy in immobilization of one of the limbs, ET is more effective, because it could produce greater increases in muscle strength and muscle mass.

Andrushko et al. (37) in their review article proposed that the term of “sparing effects of cross-education” should be used for the effect of training of nonimmobilized muscle on immobilized muscle. Using functional magnetic resonance imaging, Farthing et al. (38) observed a significant increase in contralateral motor cortex activation following 3-wk of the isometric handgrip contraction training (2 sets of 8 repetitions per day; 5 d·wk^−1^) of the nonimmobilized forearm. They concluded that the sparing effect was associated with increased motor cortex activation. Pearce et al. (34) observed maintenance in ipsilateral, untrained, corticospinal excitability assessed by transcranial magnetic stimulation when a traditional resistance (concentric–eccentric) training (4 sets of 6–8 repetitions per day; 3 d·wk^−1^) was performed by the elbow flexors during a 3-wk of sling immobilization (15 h·d^−1^). It is possible that these are produced greater by ET than CT. Importantly, the present study found slight but significant increases in MVCiso and MVCcon torques for the immobilized arm in the ET group (Table 1). It is perplexing how this was caused.

It is also not known how muscle atrophy by immobilization was completely abolished by eccentric-only training (Table 1). Phillips and McGlory (39) reported that the primary mechanism of atrophy from disuse or immobilization was due to a decreased rate in protein synthesis rather than increased protein breakdown. Andrushko et al. (10) showed that CSA of an immobilized forearm was preserved by ET of the wrist flexors performed three times a week (1.3%), whereas the CSA was reduced by the 4-wk immobilization without training (−2.3%). However, in the study by Andrushko et al. (10), no CT group was included; thus, it is not known whether ET is superior to CT for the sparing effects. Hendy and Lamon (40) speculated that the regulation of the forkhead box protein O 1/3 (FOXO1/3), mitogen-activated protein kinase (MAPK), and 5′ adenosine monophosphate-activated protein kinase (AMPK) might play a role in the contralateral sparing effects, but none have empirically tested these yet. It may be that the ET of the nonimmobilized arm maintained the muscle protein synthesis of the immobilized arm in the present study. It should be noted that this was achieved by relatively small volume of eccentric contractions in the present study (six sessions over 3 wk; the number of eccentric contractions was 30, and the average training load was 72 ± 15 kg per session). It is important to note that the sparing effects of cross-education can be obtained by a small volume of ET. The underpinning mechanisms of the sparing effects are warranted to be investigated further.

### Effects of ET versus CT on muscle damage of the immobilized arm after 30EC

To the best of our knowledge, this was the first study to investigate the cross-education effect of resistance training during immobilization on muscle damage induced by a bout of maximal eccentric exercise of the immobilized arm. The changes in the indirect muscle damage markers after 30EC in the control group did not seem to be largely different from those after 30EC of the elbow flexors performed by sedentary individuals without immobilization reported in previous studies (9,14). However, it might be that the magnitude of eccentric exercise–induced muscle damage was exacerbated by immobilization, which should be investigated further. When compared with the control group, the changes in MVCiso torque, ROM, muscle soreness, and plasma CK activity after 30EC were significantly smaller for the ET and CT groups (Fig. 4). This shows that the ET and CT conferred muscle damage protective effects on the contralateral arm. Importantly, the changes in the variables of the ET group were smaller, and recovery of MVCiso torque and ROM was faster, when compared with the CT and control groups. As shown in Figure 5, the magnitude of the contralateral protective effect of the ET group (average, 83%) was significantly greater than that of the CT group (43%). This suggests that the muscle damage was better protected by the ET than CT of the nonimmobilized arm.

It is important to note that the CT of the nonimmobilized arm seems to confer muscle damage protective effect on the contralateral (immobilized) arm. Tseng et al. (9) showed that a 5-wk of progressive concentric elbow flexor training did not attenuate muscle damage induced by a subsequent bout of maximal eccentric exercise of the homologous muscle of the opposite arm. Thus, it is interesting that the CT reduced muscle damage induced by 30EC by the immobilized arm to some extent (Figs. 3, 4). This may be associated with the preservation effect of muscle strength and size of the immobilized arm by the CT of the nonimmobilized arm as discussed previously. It is possible that stimulus by the cross-education effect to the immobilized arm by the CT attributed to the muscle damage attenuation.

Regarding the muscle damage protective effect by ET, we previously reported that the contralateral repeated bout effect was evident when the second bout of maximal eccentric contractions of the elbow flexors was performed by the contralateral arm of the first bout at 1 d (changes in muscle damage markers were attenuated by 51% in average), 1 wk (48%), or 4 wk (26%), but not at 0.5, 6, or 12 h or at 8 wk later (14). It has been shown in the previous study (41) that muscle damage induced by maximal eccentric exercise was attenuated or abolished by preconditioning exercises performed by the same and opposite homologous muscle. The new finding in the present study was that this was also induced by the progressive resistance exercise (six sessions) of the contralateral arm. The magnitude of the contralateral protective effect conferred by the ET (average of MVCiso, ROM, and muscle soreness; 83%) in the present study (Fig. 4) seems to be greater than that of the contralateral repeated bout effect reported in the previous study (14) reporting that the effect was 69% for 1-d and 52% for 7-d interval between bouts.

Hyldahl et al. (41) have documented that the repeated bout effect is induced by a combination of neural adaptations, muscle–tendon complex behavior changes, extracellular matrix structural remodeling, and modified inflammatory responses. It seems likely that the contralateral protective effect is related more to neural and modified inflammation adaptations, although adaptations at muscle–tendon complex and extracellular matrix should be not ruled out. It may be that the underlying mechanisms of the contralateral protective effect are somewhat similar to those of the cross-education effect in which a resistance training of one limb increases muscle strength of the contralateral limb (9–11). As described previously, the sparing effects of cross-education may be potentially elicited to the muscle damage protective effect. It is possible that adaptations at the cortical and spinal levels were involved in the contralateral muscle damage protective effect conferred by the ET and CT. Kidgell et al. (33) showed that the extent of the cross-education effect on MVCiso of the wrist flexors was significantly greater after ET (+47%) than CT (+28%) performed three times a week for 4 wk. They also showed that ET modulated corticospinal excitability and inhibition of the untrained limb to a greater extent than CT. It seems possible that the effects of eccentric contractions performed by the nonimmobilized arm were transferred to the contralateral immobilized arm greater. In a review article, Hendy and Lamon (40) proposed that functional reorganization of the motor cortex would facilitate the effects of cross-education, and cross-activation of the “untrained” motor cortex (ipsilateral to the trained limb) by increased neural drive from the “untrained” motor cortex contributes to the cross-education effect. These may be associated with the contralateral muscle damage protective effect observed in the previous study (9).

As for the inflammation or systemic factors, it has been documented that modified inflammatory or systemic factors play a role in the contralateral repeated bout effect (41,42). Xin et al. (42) reported that an increase in inflammatory-related transcription factor nuclear factor κ–light-chain-enhancer of activated B cells (NF-κB) after the second bout was significantly attenuated not only in the vastus lateralis that was used in the maximal eccentric contractions of the ipsilateral knee extensors (123% ± 3%; relative to the control leg without exercise) but also in the opposite leg that was not used in the exercise (109% ± 3%). Because the NF-κB is an effector of an upstream mechanistic pathway, it could be transferred to the nonexercising muscles (42). Thus, this might be associated with the contralateral muscle damage protective effect found in the present study. As shown in Table 1, the ET reduced muscle hardness (muscle became more compliant) not only in the trained arm but also in the immobilized arm. It is not known how this happened, but it has been shown that compliant muscles are less susceptible to eccentric exercise–induced muscle damage (43,44). Thus, it may be that the immobilized arm of the ET group was more resilient to mechanical strain in 30EC. More studies are warranted to investigate the mechanisms underpinning the muscle damage protection including the contralateral one.

### Limitations of the present study

The current study has several limitations. First, only young sedentary men were used as participants; thus, it is not known whether other populations such as women, elderly and fragile individuals, or people with chronic diseases respond similarly. Second, the current study did not monitor physical activity of the immobilized arm during the experiment. Third, the present study did not include eccentric strength measures, because eccentric strength measures would affect the adaptations (12,32). However, it is interesting to investigate how eccentric strength changes with the ET, CT, and control condition in a future study. Fourth, EMG was only taken from the biceps brachii during the MVCiso torque measures, and EMG activity was not normalized in the present study. Fifth, the results of the present study could not be generalized to other muscles such as leg muscles (e.g., knee extensors and flexors) as mentioned previously. Fifthly, because of the relatively small sample size for the correlation analyses (*n* = 12 per group), the interpretation of the correlation results needs to be confirmed in a study with a larger sample size. Lastly, the current study was rather descriptive, and a mechanistic approach (e.g., biopsies, measures of protein regulation) to examine the possible mechanisms underpinning the effects was not investigated.

### Practical significance and future research directions

The findings of the current study provide some useful information for prevention of muscle strength loss and atrophy by immobilization, and attenuation of muscle damage by resistance exercise after immobilization. To minimize the negative effects of immobilization, resistance training using eccentric contractions of the nonimmobilized arm can be recommended. It is important to investigate further if the findings of the present study are replicated for other muscles such as elbow extensors, knee extensors, and plantar flexors.

It is necessary to investigate whether eccentric resistance training of nonimmobilized muscles is effective for attenuating or maintaining the negative effects of immobilization in real injuries such as ligament sprains or tears, bone fracture, and postsurgery (e.g., joint replacement, anterior cruciate ligament) that accompany inflammation. Previous studies (45–50) have shown that the cross-education effects are still observed in musculoskeletal injuries when a nonaffected limb receives a resistance training, but ET was not used in these studies. It is also interesting to apply the contralateral eccentric resistance training to a less impaired limb for patients with stroke, as two studies showed that resistance exercise training of a less impaired limb provides positive effects on an impaired limb (48,49). In the present study, the immobilization period was limited to 3 wk, and the training of the nonimmobilized arm was performed twice a week. However, the immobilization period could be longer, and there are many possible training protocols. Thus, further studies are warranted to identify the most effective resistance training protocols to the nonimmobilized limb.

The present study showed that muscle damage after maximal eccentric exercise of the immobilized arm was attenuated by ET or CT of the nonimmobilized arm. However, it is not likely that immobilized muscles perform maximal eccentric contractions right after immobilization. To minimize muscle damage, the intensity of eccentric contractions should be increased gradually from low intensity over sessions. Thus, if one of the limbs is immobilized or not utilized for a while, it is recommended that the rehabilitation of the immobilized muscles is started from low-intensity exercise (e.g., 10% 1RM) even if nonimmobilized muscles receive resistance training during immobilization.

## CONCLUSIONS

In conclusion, ET of the nonimmobilized arm was effective for eliminating the negative effects of immobilization. When the immobilized arm performed 30EC after 3-wk ET of the trained arm, the magnitude of muscle damage was attenuated by contralateral ET and CT, but it is important to know that ET has a potent training and contralateral protective effect than CT. These results suggest that eccentric resistance training of the nonimmobilized limb muscles provides beneficial effects on the immobilized limb muscles.
